# Development of Carbamazepine Nanostructured Lipid Carrier Loaded Thermosensitive Gel for Intranasal Delivery

**DOI:** 10.34172/apb.2021.016

**Published:** 2020-11-07

**Authors:** Sanjeevani Shekhar Deshkar, Monali Shivaji Jadhav, Satish Vasudeo Shirolkar

**Affiliations:** ^1^Department of Pharmaceutics, Dr. D. Y. Patil Institute of Pharmaceutical Sciences and Research, Pimpri, Pune, India – 411018.

**Keywords:** Carbamazepine, Nanostructured lipid carrier, *In-situ* gel, Nasal permeation, Flux, Box-Behnken design

## Abstract

***Purpose:*** The present research work was designed to formulate and evaluate carbamazepine (CBZ) loaded nanostructured lipid carrier (NLC) based *in-situ* gel for nasal delivery.

***Methods:*** The NLC formulation of CBZ was prepared by microemulsion technique followed by probe sonication and evaluated for particle size, zeta potential, entrapment efficiency and in vitro drug diffusion. NLC formulation was incorporated into* in-situ* gelling formulation using poloxamer 407 (P407), poloxamer 188 (P188) and mucoadhesive polymer. The effect of concentration of poloxamer 188 (X_1_ ), type of mucoadhesive polymer (X_2_ ) and concentration of mucoadhesive polymer (X_3_ ) on gelling temperature (Y_1_ ) and drug diffusion after 8 h (Y_2_ ) was studied using Box-Behnken design. *In vivo* anticonvulsant activity of optimized formulation was studied in Wistar rats by maximal electro-convulsion model (MES).

***Results:*** The optimized CBZ NLC formulation, with 20% drug loading, 0.5:1 as Precirol:Capmul MCM ratio as lipid phase and 1:3 as Lipid:Smix ratio, resulted in 89.73±0.2% drug entrapment, 55.95±1.09% of drug diffusion after 8 h, particle size of 132.8 nm with polydispersity index of 0.302 and zeta potential of -29.2±6.1 mV. The *in-situ* gel formulation with 20% P407, 5% P188 and 0.2% chitosan was optimized and demonstrated excellent gelling ability, gelling temperature in the range of 30 to 35°C, 42.46% of drug diffusion in 8 h by Fickian diffusion mechanism and 31.34±0.76% of drug permeation through sheep nasal mucosa.* In vitro* anticonvulsant activity in MES model in rat demonstrated significant efficacy (71.95% protection against seizure in extension phase) as compared to plain *in-situ* nasal gel (50.26% protection against seizure in extension phase).

***Conclusion:*** NLC based *in-situ* gelling formulation demonstrated its potential for nasal delivery of CBZ with improved anticonvulsant activity.

## Introduction

Epilepsy is a set of chronic neurological disorder of brain characterized by a long term risk of recurrent seizure.^1^ About 50 million people in the world are affected by epilepsy and most of the population is from developing countries.^2^ Although, epilepsy cannot be completely cured but its symptoms can be controlled with the treatment of antiepileptic drugs.^3^ Carbamazepine (CBZ) is one of the anticonvulsant and antiepileptic drug used primarily for the treatment of epilepsy, neuropathic pain, and is a second line agent for the treatment of bipolar disorder and in some cases of schizophrenia. It is a sodium channel blocker, which after binding to sodium channel reduces its activity and stabilizes the brain cells.^4^ CBZ undergoes metabolism by microsomal enzyme, CYP3A4 in liver and is also responsible for auto-induction. The increased concentration of CBZ in liver induces CYP3A4 system that results in its own metabolism. This eventually leads to increased drug clearance and reduction in the half-life of drug.^5^ The fluctuation in the plasma level and narrow therapeutic index could result in various side effects of CBZ.^6^

For effective treatment of epilepsy, the drug levels in brain are important. Targeting drug to the brain could result in enhanced drug concentration in brain, thus minimizing side effects.^7^ The targeting by oral route is limited due to failure in appropriate absorption of certain drugs, first pass metabolism and failure in drug passage through blood brain barrier (BBB).^7^ Presence of BBB makes the treatment of CNS disorder very challenging and difficult. ^3^ BBB is characterized by presence of tight junctions restricting the movement of molecules by paracellular transport, thus, facilitating the drug movement only by transcellular transport, carrier mediated transport and receptor mediated endocytosis.^8^ The drug uptake in brain is further regulated by highly expressed efflux transporter, P-glycoprotein.^9^

An alternative approach of brain targeting is nose to brain delivery that enables delivery of drug through various pathways such as, olfactory pathway, trigeminal nerve pathway, cerebrospinal fluid pathway, perivascular pathway etc. Enhanced nose to brain targeting is achieved by various strategies including increase in the nasal residence time using mucoadhesive polymer, application of nanocarrier for improved drug permeation and controlled release, use of permeation enhancers and enzyme inhibitors.^9^ The smaller sizes of nanoparticle enable them to diffuse quickly by transcellular transport resulting in rapid onset of action, improved bioavailability and enhanced distribution in the target tissue based on the nature of the carrier. Drug release can be controlled using nanoparticles and also protection against enzymatic degradation is achieved.^10^

Nanostructured lipid carrier (NLC) is extensively studied nanocarrier system and consisted of a blend of solid and liquid lipid. NLC is reported to overcome the limitations of solid lipid nanoparticles (SLN) such as lower drug loading capacity and exclusion of entrapped drug during storage.^11^ Literature suggested the potential benefits of lipid nanocarrier system for nose to brain delivery. Some of the benefits are closer contact with olfactory epithelium due to smaller particle size, improved mucosal permeation, protection from P-glycoprotein efflux, drug release in a controlled fashion, higher stability with protection from microsomal enzyme and direct transport by other pathways in case of size less than 200 nm.^12^ In order to overcome the rapid mucociliary clearance, nanoparticle systems can be further incorporated into *in-situ* gel systems. *In-situ* gelling formulation of thermosensitive polymers, poloxamer 407 and 188, is extensively explored for nasal delivery. In addition to this, use of mucoadhesive agents like chitosan is reported to increase the residence time along with increasing the drug transport across mucosa.^13^

In the present study, NLC of CBZ was prepared by microemulsion method followed by probe sonication.^14^ NLC was further formulated as *in-situ* gel with poloxamer as thermosensitive polymer in combination with three different mucoadhesive polymers chitosan, hydroxyl ethyl cellulose (HEC) and hydroxyl propyl methyl cellulose (HPMC). The effect of these mucoadhesive polymers and different combinations of poloxamer 407 and 188 on characteristics of *in- situ* gel was evaluated by design of experiment. The developed NLC loaded *in-situ* gelling formulation was assessed for *in vitro* characteristics and *in vivo* anticonvulsant activity.^15^

## Materials and Methods

### Materials

CBZ was received as gift sample from Amol Organics Pvt Ltd, Vadodara, India. Precirol ATO 5, Capryol 90 and glyceryl monostearate (GMS) were generously gifted by Gattefosse India Pvt Ltd, Mumbai, India and Capmul MCM, Captex 500, Captex 200 and Caprol PGE (polyglycerol ester) were gifted by Abitech, USA. Span and tween 20 were purchased from Loba Chemie, Mumbai, India. Poloxamer 407 (Kolliphor P 407) and Poloxamer 188 (Kolliphor P 188) were received from BASF, Mumbai, India. Hydroxyethyl cellulose (Natrosol 250) and hydroxy propyl methyl cellulose, low viscosity (Methocel E50 LV) were received from Ashland, Mumbai, India and Colorcon Asia Pvt Ltd, Mumbai, India, respectively.

### Method

#### Selection of lipid and oil

In order to select the lipid and the oil phase for NLC preparation, solubility study of CBZ in different lipids and oils was performed. To achieve maximum loading, drug should demonstrate high solubility in lipid and oil. The solubility of CBZ in lipids, Compritol, Gelucire, Precirol and GMS, was determined by heating lipids (1 g) above their melting point and adding small increments of drug in these lipids. The solubility of drug was determined by visually observing lipid till its saturation point, identified by turbidity. For solubility in oils such as Caproyl 90, Capmul MCM, Captex 200, Captex 500 and Vaprol, CBZ was added in small increment to 2 mL of oil with constant stirring till saturation exists. The mixture was kept at 25 ±0.5°C at 100 rpm in a water bath shaker (Neolab, India) for 72 hours in order to attain the equilibrium. After 72 hours, the solutions were centrifuged at 3000 rpm for 15 minutes and supernatant was appropriately diluted with methanol to analyse the CBZ at 284 nm using UV spectrophotometer (Shimadzu, UV-1800, Japan).

#### Preparation of nanostructured lipid carrier (NLC) of CBZ

CBZ NLC was prepared by microemulsion technique followed by probe sonication.^16^ To the Molten Precirol, Capmul MCM was added at 80°C followed by addition of CBZ. Surfactant mixture of tween 80 and span 20 was added to the lipid mixture with continuous stirring. A clear microemulsion was obtained after addition of specific amount of hot aqueous phase along with butanol to the lipid phase at the same temperature (80°C) .The microemulsion was further poured into external aqueous phase, in a ratio of internal to external phase as 1:10, at 4°C with mechanical stirring for 1 hour. This was followed by probe sonication of NLC dispersion for 15 minutes. The effect of CBZ concentration, ratio of Precirol:Capmul MCM, ratio of lipids to surfactant mixture and sonication time on responses, particle size and drug entrapment in NLC was studied (Table 1).^15^

**Table 1:** Optimization of CBZ NLC Formulation

**Code**	**Dilution ratio**	**Lipid: Smix**	**Precirol : Capmul MCM ratio in lipid phase**	**Drug concentration in lipid phase** **(%)**	**Entrapment** Efficiency ( %)*	Particle size (nm)*	PDI*
F1	1:10	1:3	2:1	10	68.0±0.21	139.1±2.5	0.357±0.1
F2	1:25	1:3	2:1	10	84.0±0.11	149.4±3.2	0.197±0.2
F5	1:25	1:2	2:1	10	76.76±0.50	346.9±5.0	0.379±0.1
F6	1:25	1:4	2:1	10	86.42±0.22	154.9±2.2	0.239±0.2
F7	1:25	1:3	9:1	10	75.88±0.22	271.0±6.0	0.379±0.3
F8	1:25	1:3	4:1	10	77.91±0.10	255.0±4.2	0.421±0.2
F3	1:25	1:3	2:1	20	89.73±0.33	149.4±2.3	0.323±0.1
F4	1:25	1:3	2:1	30	87.0±0.22	151.0±3.5	0.379±0.2

*Values are expressed as Mean ± SD (n=3), CBZ NLC: Carbamazepine loaded nanostructured lipid carrier

For the purpose of solid state characterization, CBZ NLC dispersion was lyophilized using a freeze dryer (Martin Christ, Alpha 1-2 LD Plus, Germany) in presence of trehalose as cryoprotectant (3% w/v). After freezing at -20°C for 12 hours, NLC dispersion was dried at -50°C at 0.1 mbar pressure for 24 hours.

### Characterization of NLC 

#### Entrapment efficiency

Entrapment efficiency (EE) was determined by dialysis bag method. NLC dispersion (2 mL) was filled in dialysis bag with both ends tied. The bag was placed in 50 mL mixture of water and methanol (3:1) at 37±0.5°C and stirred at 100 rpm on a magnetic stirrer. After 30 minutes, 5 mL of sample was removed from the beaker, filtered using a syringe driven filter (0.45 µ) and analysed for drug content by UV spectrophotometer (Shimadzu, UV-1800, Japan) at 284.0 nm.^17^ The analysed drug was considered as free and the drug entrapment was calculated using following formula.

$$EF\%=\frac{Total\;CBZ-Unetraped(free)CBZ}{Total\;CBZ}\times 100$$

#### Drug content

The NLC (equivalent to 5 mg CBZ) was dissolved in warm methanol in order to dissolve drug and lipid, magnetically stirred for 10 minutes and sonicated for 5 minutes. The CBZ content in methanol was analysed after membrane filtration (0.45 µ) using UV spectrophotometer at 284 nm against methanol as blank.^18^

#### Particle size and zeta potential measurement

The particle size of CBZ NLC was determined using dynamic light scattering (Horiba, SZ 100, Japan). NLC dispersion was diluted with distilled water and ultrasonicated for 10 minutes followed by measurement at fixed angle 90° at 25°C carried out in triplicate. For zeta potential analysis, the sample was diluted by conducting solution and zeta potential was measured (Horiba, SZ 100, Japan).^19^

#### Transmission electron microscopy (TEM)

To confirm size and shape of nanoparticle, TEM (Philips, CM 200, Germany) was performed. NLC dispersion was diluted with distilled water prior to analysis. The sample (5 µL) was placed on copper grid coated with carbon. Sample was stained with 20 µL of 2%w/v of phosphotungstic acid. The grid was allowed to dry. The observations were made at 200 kV acceleration voltage and 2.4 A_0_ resolution.^20^

#### Fourier-transform infrared spectroscopy (FTIR)

In order to study the interaction between drug and lipid, IR absorption spectra of CBZ, Precirol and lyophilised NLC with cryoprotectant were recorded by potassium bromide dispersion technique in which dry samples and potassium bromide were placed in sample holder and infrared spectrum was recorded using FTIR Spectrophotometer (Shimadzu, 8400S, Japan) over a range of 400-4000 cm^-1^.^21^

#### Differential scanning calorimetry (DSC)

For study of physical state of CBZ in SLN formulation, the CBZ, its physical mixture with excipients and lyophilized NLC were subjected to DSC analysis (PerkinElmer, 4000, USA). Accurately weighed 1 mg sample was placed in a sealed aluminium pan, and the sample was heated under nitrogen flow (20 mL/min) at a scanning rate of 10°C per min from 30 to 350°C. An empty aluminium pan was used as reference.^22^

#### X-Ray diffraction (XRD)

XRD (Rigaku, Miniflex 600, Japan) was performed using Cu K 2α rays with a voltage of 40 kV and a current of 15 mA. Samples were scanned for 2θ from 20 to 80°. Diffraction patterns for CBZ, its physical mixture with, trehalose and lyophilized NLC formulation with trehalose were obtained.^23^

### Preparation of NLC loaded in-situ gel

NLC loaded *in-situ* gel of CBZ were prepared by using cold method. The required amount of P 407(20% w/w) and P 188 were dispersed in NLC preparation containing benzalkonium chloride (0.01% w/w) at 5±2°C. The poloxamer solutions were stored in the refrigerator for 24 hours until polymer completely dissolved in NLC preparation. For obtaining *in-situ* gelling formulations with mucoadhesive polymers, viz. HEC, HPMC, and chitosan, the procedure was slightly modified. For HPMC E 50 and HEC formulations, the polymers were dispersed in the NLC preparation with continuous stirring till the dissolution. For chitosan formulation, chitosan was first dissolved in 1% acetic acid. To the NLC dispersion, P 407 and P 188 with benzalkonium chloride were added and refrigerated for 24 to 48 hours. Chitosan solution was added to poloxamer NLC dispersion with stirring to obtain a clear preparation.^24^

### Experimental design

To study the effect of formulation parameters on *in-situ* gelling formulations, a 3 factors and 3 levels Box-Behnken design was applied. The effect of independent variables, concentration of poloxamer 188 (X_1_), type of mucoadhesive polymer (X_2_) and concentration of mucoadhesive polymer (X_3_) on responses, gelation temperature (Y_1_) and CBZ release after 8h (Y_2_) was studied. Table 2 indicates the levels of independent variables, X_1_, X_2_ and X_3_. The factor X_2_ was categorical factor in which different mucoadhesive polymers, viz. HPMC (-1), HEC (0) and chitosan (+1) were used in formulations. The data from 17 trial runs was analyzed using Design Expert software (Stat Ease, version 9.0, USA). The contour plots and 3D surface response plots were generated in order to study the influence of independent variables on responses.

**Table 2:** Box-Behnken design for preparation of CBZ NLC *in-situ* gel

**Batch**	**X**_1_^a^	**X**_2_^b^	**X**_3_^c^
F1	-1	0	-1
F2	0	0	0
F3	-1	0	1
F4	-1	-1	0
F5	1	0	-1
F6	0	-1	-1
F7	0	0	0
F8	0	1	1
F9	0	0	0
F10	-1	1	0
F11	1	0	1
F12	1	-1	0
F13	0	-1	1
F14	1	1	0
F15	0	1	-1
F16	0	0	0
F17	0	0	0

^a^X_1_= Concentration of poloxamer 188 (-1 = 0% w/w, 0 = 5%w/w, 1 = 10% w/w), ^b^X_2_=Type of polymer = (-1 = hydroxy propyl methyl cellulose, 0 = hydroxyl ethyl cellulose, 1 = Chitosan), ^c^X_3_= Mucoadhesive polymer concentration (-1= 0.2% w/w, 0= 0.4% w/w, 1= 0.6%w/w), CBZ: Carbamazepine, NLC: Nanostructured lipid carriers

### Evaluation of in-situ gel

#### Gelling ability

For determining the gelling ability, 100 µL of gel was added in 2 mL of phosphate buffer (pH 7.4) contained in a glass vial maintained at 37°C.^24^ The phase transition of formulation from sol to gel was observed and numerical scores were assigned. The grading was done as, no phase transition (-), gel formation after 60s and collapsed rapidly within 1 hour (+), gel formation after 60 s and gel collapsed within 3 hours (++) and gel formation within 60 s and stable for more than 6 hours (+++). The mean of the three readings was recorded.^25^

#### Gelation temperature

In order to determine gelling temperature, *in-situ* gelling formulation (5 g) was placed in glass vial placed on magnetic stirrer. The solution was heated slowly with constant stirring at 100 rpm.^24^ The temperature at which magnetic bead stopped moving was noted as the gelling temperature.

#### Effect of dilution on gelling temperature

After administration into nasal cavity, gel formulation may get diluted due to presence of nasal secretion. In order to study the effect of dilution on gelling temperature of *in-situ* gelling formulations, phosphate buffer pH 7.4 was added (0.25 mL/g gel) to 4 g of formulations in a vial. The gelling temperature of the diluted formulation was determined using previously described method.^26^

#### Drug content

To determine the drug content, 1mL of *in-situ* gel was added to 100 mL of methanol. The solution was filtered and analyzed by a UV spectrophotometer (Shimadzu, UV-1800, Japan) at λ_max_of 284 nm. For each formulation, the experiment was repeated thrice.^27^

#### Viscosity

The viscosity of *in-situ* gel was determined at 37°C using Brookfield viscometer (Brookfield, RVDV pro II, USA) using T bar spindle with Helipath attachment to obtain the viscosity at 0.5, 2, 5, 10, 20, 50 and 100 rpm.

#### Spreadability

The spreadability of *in-situ* gelling formulation was determined using a fabricated apparatus consisting of a glass slide that is mounted on a triangular shaped glass box. The slope of the triangular box was making an angle of 45° to the horizontal surface of box. The slide was attached to the slanting surface and the complete assembly was maintained at 37°C. The formulation was placed on slanting glass slide and distance travelled by the liquid formulation before converting to gel was observed as its ability to spread.^28^

#### Mucoadhesive strength

In order to quantify the mucin-polymer interaction, the mucoadhesive strength of *in-situ* gelling formulations was measured using goat nasal mucosa by texture analyzer (Brookfield, CT3 Texture Analyzer, USA). Goat nasal mucosa was collected from the slaughter house. After separation from the underlying tissue, the nasal mucosa was washed several times with saline solution. The tissue was rapidly frozen and stored at -20°C. The mucosa was thawed at room temperature before testing, hydrated with phosphate buffer pH 6.8 and placed on the base of the texture analyzer. Preformed gels at 37°C were applied to the probe of texture analyzer with the help of adhesive tape. The contact of probe of texture analyzer was made with the tissue for 20 s with contact load of 50 g. After establishing proper contact, the probe was withdrawn at a rate of 1 mm/s. The display reading showed the force required to detach gel formulation from the nasal tissue and noted as mucoadhesive strength (g).^29^

#### In vitro diffusion

*In vitro* diffusion was carried out using Franz diffusion cell assembly. Dialysis bag (10 to 12 kDa cut off) was soaked in phosphate buffer pH 7.4 for 24 hours before experiment. The diffusion cell assembly consisted of three diffusion cells with donor and receptor compartment and placed in a rectangular water bath at 37°C. The receptor compartment (50 mL capacity) was filled with phosphate buffer pH 7.4. The magnetic stirring was provided to the receptor fluid as well as to the outside water bath. The dialysis membrane was fastened between the donor and receptor compartment and 2 g of gel was placed on donor side. After every hour, the aliquot of receptor fluid was removed, suitably diluted and concentration of drug was analysed using UV spectrophotometer (Shimadzu, UV-1800, Japan) at 284 nm. In order to maintain sink condition, the volume of receptor fluid was maintained by introducing the same amount of fresh buffer.^29^

#### Ex vivo diffusion through sheep nasal mucosa

To determine diffusion of drug through nasal mucosa, freshly excised sheep nasal mucosa was collected from the slaughter house and rinsed with saline solution. Cartilages were removed properly, and the mucosal membrane was isolated and washed several times with phosphate buffer (pH 7.4). *Ex vivo* drug diffusion study was performed using a Franz-type diffusion cell assembly. The membrane was fastened between donor and receptor compartments of diffusion cell. The receptor compartment was filled with 50 mL of fresh phosphate buffer (pH 7.4). *Ex vivo* diffusion of CBZ NLC *in-situ* gel and gel without NLC was conducted by placing 2 g of *in-situ* gel onto the sheep nasal mucosal membrane. The samples from the receptor phase were withdrawn at periodic time intervals, analysed using UV–visible spectrophotometer at 284 nm. The amount of drug diffused was calculated from the calibration curve of CBZ in phosphate buffer (pH 7.4).^30^

#### In vivo anticonvulsant activity

To evaluate the efficacy of developed formulation by nasal route, *in vivo* anticonvulsant activity of CBZ NLC loaded *in-situ gel* was assessed in Wistar rats and compared with the activity of nasal CBZ plain *in-situ* gel and oral CBZ dispersion. Healthy and convulsion free male Wistar rats, weighing 200–220 g, were used for the study. The animals were grouped in four groups with six in each. Group I was kept as control without any treatment. CBZ oral dispersion was administered to group II (133µg/kg). In group III and IV, CBZ *in-situ* gel and CBZ NLC based *in-situ* gel (133 µg/kg of CBZ) were administered to the rats intra-nasally with the help of syringe and the rats were held in supine position for 1 min. At time 0.5, 1, 2, 3 and 4 hours after administration of formulations, the animals were given maximal electroshocks of 150 mA for 0.2 seconds to the cornea by using electro-convulsometer. Animals that showed hind limb tonic extensor phase represented the maximal seizure activity and its duration was taken as a measure of the spread of impulse in convulsion. The various phases of maximal electroshock induced convulsion for each animal were noted from which the protection against maximal electroshock was calculated.^31^ The statistical difference in the groups was determined by applying two way analysis of variance followed by multiple comparison test (GraphPad Prism 7).

## Result and Discussion

### Selection of lipid and oil

**Figure 1:**
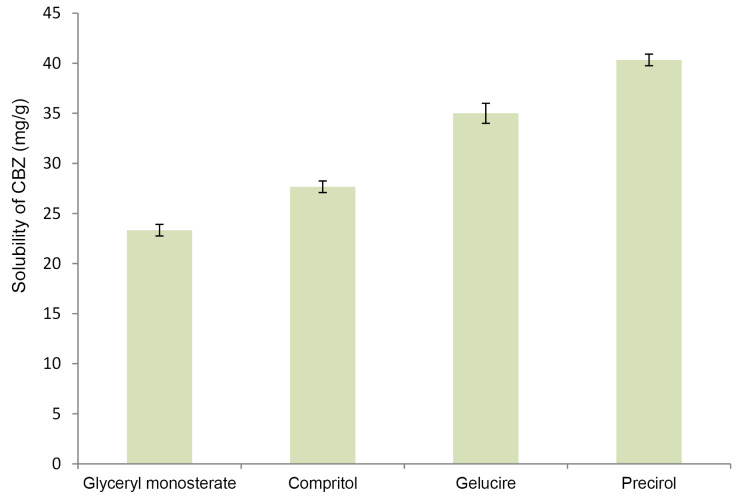
Solubility of carbamazepine in lipids (mg/g).

**Figure 2:**
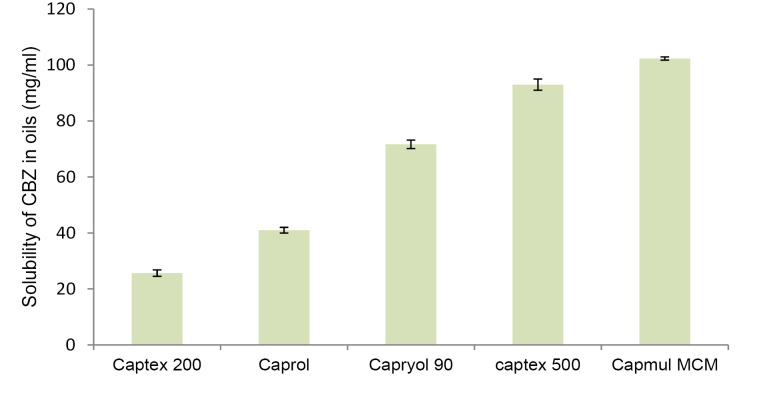
Solubility of carbamazepine in oils (mg/mL).

For achieving maximum drug loading in NLC, drug should exhibit maximum solubility in solid lipid as well as oil. Figure 1 and Figure 2 demonstrate solubility of CBZ in various lipid and oils respectively. CBZ demonstrated highest solubility in Precirol (40 mg/g) and Capmul MCM (102 mg/mL). The solubility of CBZ was higher in oil as compared to solid lipid. This suggested the possibility of higher drug loading in NLC as compared to SLN. Considering the higher solubility of drug, Precirol and Capmul MCM were selected as lipid and oil for NLC preparation.^32^

### Preparation and evaluation of NLC

CBZ NLC were prepared using microemulsion method followed by probe sonication as previously reported.^16^ Effect of different parameters (Table 1) on drug entrapment and particle size of NLC were investigated. As internal:external phase dilution ratio was changed from 1:10 to 1:25, there was significant increase in drug entrapment and also increase in particle size. When proportion of Capmul MCM (oil) in the lipid blend was increased, CBZ entrapment was also increased. This could be attributed to the disordered structure or asymmetry resulted due to mixing of two dissimilar lipids.^18^ Increasing the oil proportion in the lipid blend also resulted in lower particle size. This could be due to reduction in the possible aggregation of congealed droplets during emulsification process.^33^

Increase in the drug concentration also increased the CBZ entrapment in NLC whereas there was no significant effect on particle size. When lipid: Smix ratio was changed from 1:2 to 1:3, the drug entrapment was increased and particle size was decreased. The effect was observed due to increase in concentration of surfactant that decreased the interfacial tension between the lipid and aqueous phase, thus reducing the particle size. The change in entrapment efficiency could be attributed to the nature of surfactant that may alter the partitioning of drug in lipid and aqueous phase. With increase in surfactant concentration in the lipid phase, the CBZ partitioning into lipid phase could have increased resulting in higher drug entrapment. Further change in lipid: Smix ratio (1:3 to1:4), did not significantly alter particle size and entrapment efficiency. CBZ concentration (% of lipid phase) also had a significant impact on NLC preparation. With increase in CBZ concentration from 10% to 20%, drug entrapment and particle size was increased. Further increase in drug concentration to 30% did not reveal significant effect on entrapment. Similarly, effect on particle size was also insignificant. For further study, dilution ratio of 1:25, drug concentration of 20%, Precirol:Capmul ratio of 2:1 and lipid: Smix ratio of 1:3 (formulation F2) was selected. The polydispersity index of optimized formulation was 0.197 indicating narrow particle size distributions. NLC formulation exhibited zeta potential of -29.2 mV. Zeta potential is the measure of particle stability. The higher zeta potential value, closer to -30 mV, indicated stability of NLC dispersion. Additionally, the presence of nonionic surfactant like tween 20 in the formulation could have imparted steric stabilization to the colloidal dispersion preventing aggregation.^34^

### FTIR

**Figure 3:**
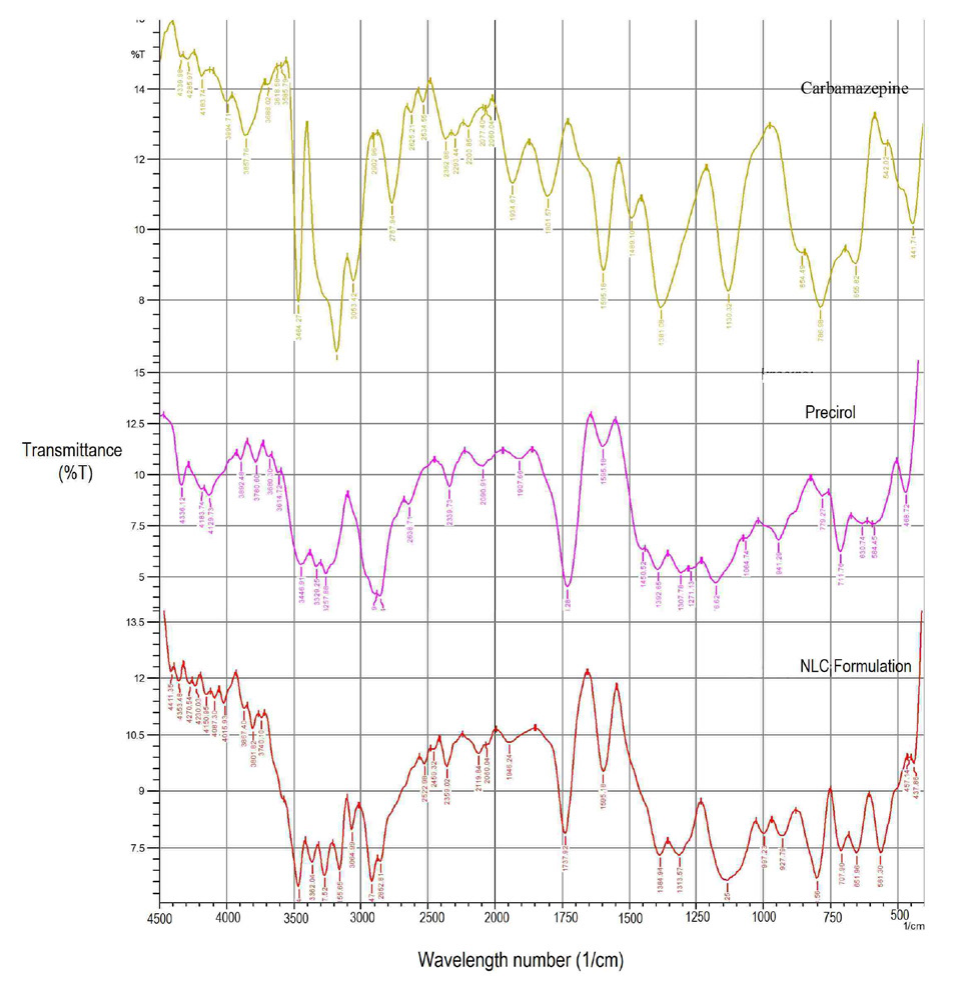
FTIR of Carbamazepine, Precirol, Lyophilized nanostructured lipid carrier formulation.

Figure 3 represents FTIR spectra of CBZ, Precirol, and CBZ NLC formulation. For CBZ, characteristics bands appear at 1489.10, 1595.18, 786, 3464, 1130.32 cm^-1^ indicating C=C stretching of aromatic ring, amide carbonyl, C-H bending of aromatic ring, aromatic –NH2, and C-N stretch.For Precirol, a broad reflectance band was observed in the range of 2854.7 to 3780.6 cm^-1^ with a maximum ~3257.88 cm^-1^, which is attributed to the OH stretching vibrations and 1750 cm^-1^ indicating carboxylic group. In FTIR spectra of CBZ loaded NLC formulation, the characteristic bands of CBZ indicating –C=O stretch and –NH2 stretch appeared at 1597.11 and 3462.34 cm^-1^ respectively. Lipids indicating a broad reflectance band in the range of 2852.81 to 3740.10 cm-1 indicated –OH stretching vibration with maximum at 3267.52 cm^-1^ and carboxylic group appear at 1737.92 cm^-1^ respectively. This indicated absence of any interaction between CBZ and lipid in NLC formulation.^35^

### TEM

**Figure 4:**
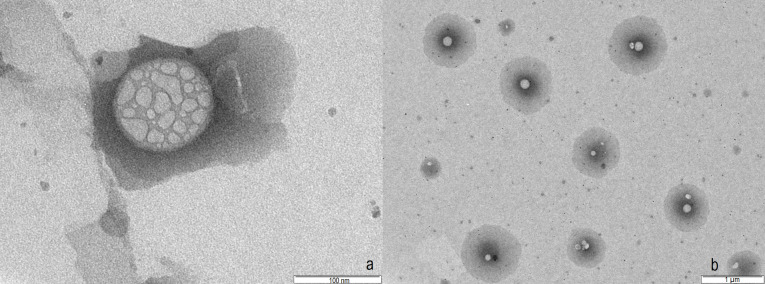
(a) and (b) shows transmission electron micrograph of carbamazepine loaded nanostructured lipid carrier.

The morphology and internal structure of NLC optimized formulation (F2) was studied using TEM (Figure 4). The TEM study revealed spherical shape of NLC in the range of 100-150 nm which is in agreement with dynamic light scattering results. The internal structure of NLC is also evident in TEM. The presence of oil nano-compartments embedded in solid lipid is clearly seen in each NLC structure.^36^

### DSC

**Figure 5:**
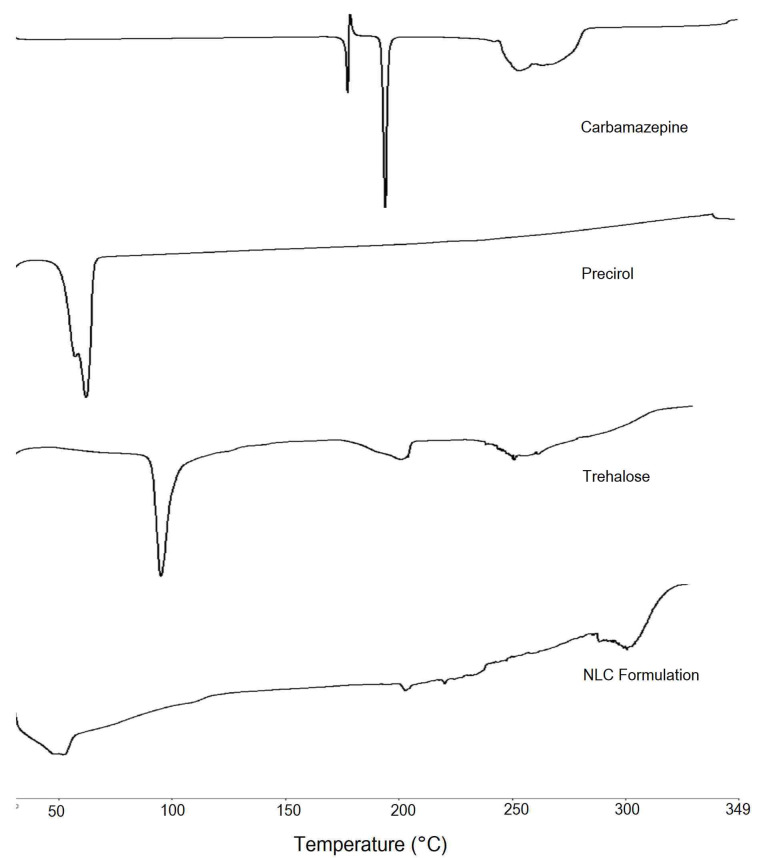
DSC of Carbamazepine, Precirol, Trehalose, Lyophilized carbamazepine loaded nanostructured lipid carrier formulation with trehalose.

For investigating the changes that occurred in the crystallinity of drug and lipid during NLC preparation, DSC study was performed on CBZ, Precirol, trehalose dihydrate as cryoprotectant and lyophilised CBZ loaded NLC formulation (Figure 5). CBZ demonstrated sharp endothermic peak at 192.71°C indicating its melting point that is in close agreement with the reported value in the literature.^37^ The DSC thermogram of Precirol showed sharp endothermic peak at 62°C indicating melting point of the lipid and its crystalline nature. DSC of trehalose dihydrate indicated endotherms at 99°C and 214.15°C representing melting point of trehalose in dihydrate and anhydrous form. DSC of formulation indicated two broad endotherms ranging from 45.8°C to 56.5°C and 200.9°C to 203.9°C representing lipid and trehalose respectively. The shift in the endotherm of lipid indicated changes in the lipid crystallinity. This could be due to solubilization of drug and oil in the crystalline Precirol leading to a change in its ordered crystalline structure. Absence of CBZ endotherm further confirms the complete drug solubilisation in lipid and entrapment.

### XRD

**Figure 6:**
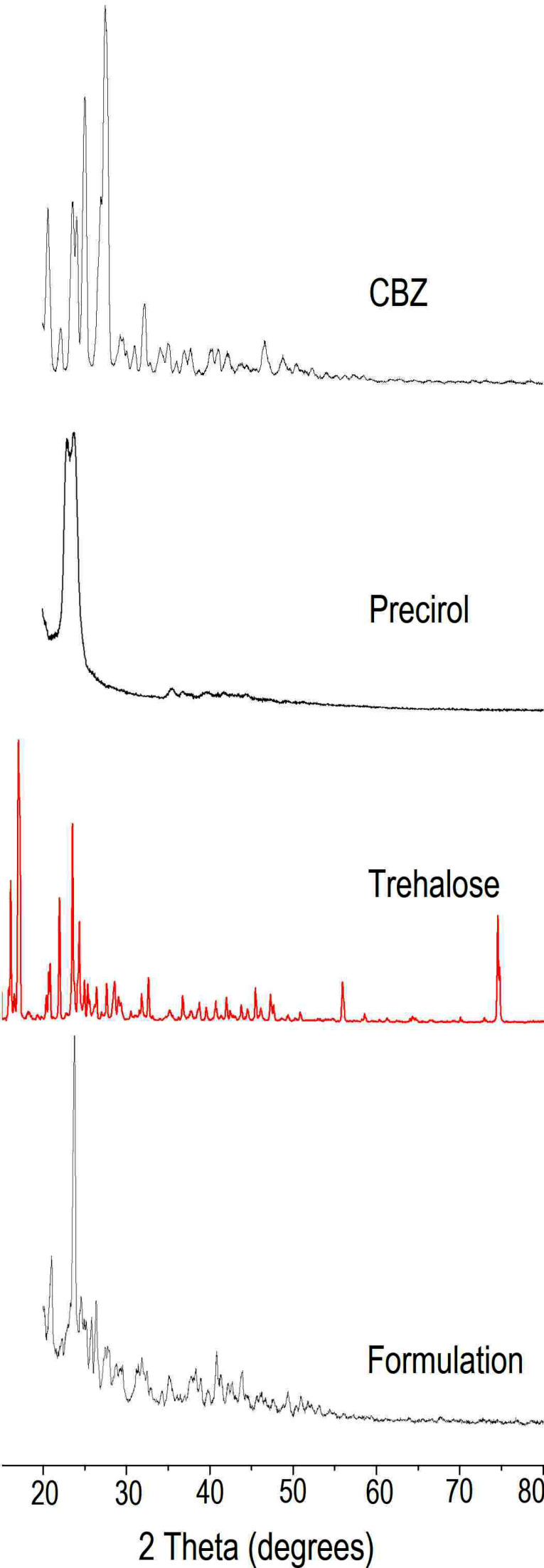
XRD of Carbamazepine, Precirol, Trehalose, Lyophilized carbamazepine loaded nanostructured lipid carrier formulation with trehalose.

The changes in the crystallinity of drug during NLC preparation was further confirmed by XRD (Figure 6). For solid state characterization, NLC dispersion was lyophilised in presence of trehalose as cryoprotectant that allowed formulation to be in powder state. XRD of CBZ indicated characteristics sharp peaks at 20.68°, 23.6°, 23.7°, 25.14° and 27.58° indicating crystalline nature of drug. Precirol demonstrated peaks in the region 22.7° to 24.08°. XRD of trehalose indicated sharp characteristic peaks at 15.06, 15.62, 15.77, 17.19, 22.09, 23.67, 24.48, 31.86, 32.73, and 45.52°. XRD of formulation indicated presence of few peaks at 20.98° and 23.78°. The peak at 20.98° indicated the CBZ peak when compared to XRD data of CBZ whereas peak at 23.78° represented Precirol and at 29.66° and 29.7° represented trehalose. The decrease in the peak intensity indicated changes in the crystallinity of lipid that is in agreement with the DSC data. The major characteristic peaks of drug were absent indicating changes in the drug during entrapment in NLC. Few low intensity peaks in the range, 20.68° to 20.98° suggested presence of drug in semi-crystalline form.

### Preparation and evaluation of in- situ gelling formulation

*In-situ* gelling formulation of the thermosensitive polymer, poloxamer, exhibits sol state at lower temperature that is easier to handle and administer. After administration to body temperature, it undergoes sol to gel transition that will allow increased contact time in the nasal cavity. Poloxamer 407 is a thermosensitive polymer with a ratio of 7:3 for polyoxyethylene oxide (PEO) and polyoxypropylene oxide (PPO) blocks. PPO blocks of poloxamer, at higher temperature, undergo self-aggregation to form tightly packed micelles resulting in a gel formulation. Preliminary trials were conducted to optimize poloxamer 407 concentrations (16% and 17%). Addition of poloxamer 407, at concentration 16% and 17% w/w, resulted in a gel that was quickly formed at gelling temperature of 24 and 21.4°C respectively. However, the gel strength of resulting gel was weaker and the gel collapsed within 1 hour. Poloxamer 407, at 18% w/w concentration, resulted in a robust formulation with higher gel strength and the gel was stable for more than 6 hours. The gelling temperature of the formulation with 18% w/w poloxamer 407 was very low (18.9°C). In order to increase the gelling temperature, poloxamer188 was added to *in-situ* gelling formulation. Higher PEO: PPO ratio in poloxamer 188 than poloxamer 407 results in increase in gelation temperature. Effect of poloxamer 188 concentration along with type and concentration of different mucoadhesive polymer (HPMC, HEC and chitosan) on gelling temperature was studied using Box-Behnken design and the results are presented in Table 3.

**Table 3:** Evaluation parameters of *in-situ* gelling formulations

**Formulation code**	**Gelation temperature before dilution (** ^o^ C )*	**Gelation temperature after dilution (** ^o^ C )*	**Gelling ability#**	**pH**	Spreadability (cm)*	**Viscosity (cp at 20 rpm)**	Drug release after 8 h ( %)*
**F1**	20.2±0.1	21.3±0.1	++	4.2	1.0±0.2	2021	46.07±0.5
**F2**	29.0±0.2	30.1±0.2	+++	4.5	2.5±0.1	2116	46.18±0.3
**F3**	24.6±0.2	25.4±0.2	+++	6.2	1.3±0.1	1416	38.57±0.2
**F4**	19.1±0.1	20.7±0.3	++	7.0	1.8±0.1	1596	46.53±0.5
**F5**	30.4±0.1	31.5±0.2	+++	4.5	3.8±0.2	1095	21.95±1.0
**F6**	31.1±0.1	32.7±0.1	+++	6.8	2.9±0.3	2020	34.99±0.2
**F7**	27.9±0.2	28.4±0.2	+++	5.9	2.0±0.2	2165	42.91±0.1
**F8**	31.7±0.1	32.4±0.2	+++	6.2	3.2±0.1	1995	38.14±0.2
**F9**	28.2±0.2	29.6±0.2	+++	4.5	2.6±0.2	1982	45.37±0.5
**F10**	20.6±0.2	21.8±0.2	+++	7.0	1.6±0.2	2591	43.85±0.2
**F11**	32.3±0.2	33.8±0.2	++	6.0	4.5±0.1	2065	30.72±0.3
**F12**	33.3±0.2	34.8±0.2	+++	5.2	4.8±0.2	1985	27.99±0.1
**F13**	26.7±0.2	27.2±0.2	+++	6.5	2.2±0.2	5623	38.57±0.5
**F14**	34.5±0.2	35.8 ±0.2	+++	5.6	5.3±0.05	2135	28.18±0.2
**F15**	32.0±0.2	33.2±0.2	+++	6.3	5.5±0.1	4659	42.46±0.3
**F16**	29.0±0.2	30.2±0.2	+++	5.3	3.3±0.2	3592	46.18±0.5
**F17**	28.2±0.2	29.7±0.2	+++	5.5	3.5±0.2	3165	45.37±0.1

*Mean ±SD (n=3) #Gelling ability: No phase transition (-), gel formation after 60s and collapsed rapidly within 1 h (+), gel formation after 60s and gel collapsed within 3 h (++) and gel formation within 60s and stable for more than 6 h (+++).

### Effect on gelation temperature 

Effect of poloxamer 188 concentrations (X_1_) and mucoadhesive polymer concentration (X_3_) on gelling temperature, in presence of HPMC, HEC and chitosan as mucoadhesive polymer, is represented in equation 1, 2 and 3 respectively. The statistical significance was established using ANOVA followed by student t test (Table 4). The equations 1, 2 and 3 are as follows:

**Table 4:** Data of ANOVA for responses

**Source**	**F value**		***P*** **value** **Prob>F**	
	**Y**_1_	**Y**_2_	**Y**_1_	**Y**_2_
Model	68.16	22.95	0.0001	0.0014
X1: Poloxamer 188 concentration	581.44	147.37	<0.0001	<0.0001
X2: Type of mucoadhesive polymer	14.35	3.03	0.0085	0.1373
X3: Mucoadhesive polymer concentration	0.38	0.009	0.5637	0.9263
X_1_X_2_	15.86	0.36	0.0068	0.7134
X_1_X_3_	2.46	17.81	0.1776	0.0083
X_2_X_3_	20.51	2.17	0.0039	0.2099
X_1_^2^	58.19	46.26	0.0006	0.0010
X_3_^2^	9.46	22.81	0.0276	0.0050

 Y_1_ =+28.30+7.10*X_1_-2.215*X_3_-0.525*X_1_*X_3_-2.488*X_1_^2^+1.00*X_3_^2^ (1)

Y_1_=+28.46+4.37*X_1_+1.47*X_3_-0.525*X_1_*X_3_-2.488*X_1_^2^+1.00*X_3_^2^(2)

Y_1_=+30.45+6.98*X_1_-0.150*X_3_-0.525*X_1_*X_3_-2.488*X_1_^2^+1.00*X_3_^2^(3)

**Figure 7:**
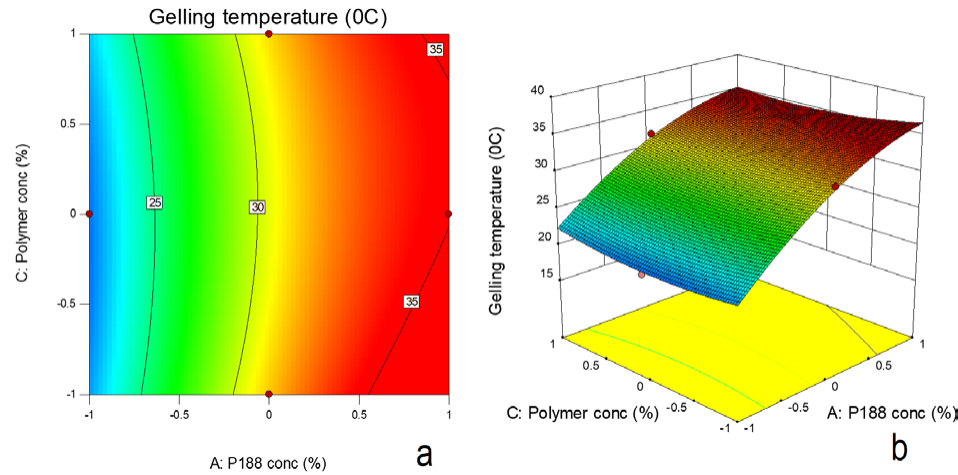
(a) Contour plot (b) Surface response plot depicting the effect of polymer concentration (chitosan and poloxamer188 concentration on gelling temperature.

In order to study the relationship that existed between independent and dependant variables, a F distribution statistics was used (Table 4). F values greater than the F critical values and low *P* values (*P* ≤ 0.05) indicated significance of the model. The effect of poloxamer 188 concentration and type of mucoadhesive polymer on gelling temperature was found to be significant (*P* ≤ 0.05) whereas effect of concentration of mucoadhesive polymer on gelling temperature was insignificant. The presence of quadratic term in the equations suggested nonlinearity in the effect. The lower difference in predicted R^2^ (0.9934) and adjusted R^2^ (0.9788) suggested suitability of the model. Figure 7a and b indicate the surface response and counter plot showing effect of poloxamer188 concentration (X1) and mucoadhesive polymer concentration (X3) on gelling temperature. In presence of different mucoadhesive polymer, increase in poloxamer 188 concentrations resulted in increase in gelling temperature. In presence of HPMC and chitosan, with increase in their respective concentration (from 0.2% to 0.6%), there was decrease in gelling temperature. This could be attributed to formation of hydrogen bond between PEO chain of poloxamer and mucoadhesive polymer, thus decreasing the gelling temperature. This was also resulted in increase in viscosity of these formulations. In case of HEC, however, increase in its concentration also increased the gelation temperature. Similarly there was decrease in viscosity of formulation with increase in mucoadhesive polymer concentration. This was unusual observation and not consistent with earlier reports. This behaviour could be the result of negative interference of HEC in poloxamer sol to gel transition. HEC gel also demonstrated lower gel strength.

CBZ NLC based *in-situ* gel showed slightly higher viscosity than CBZ based plain *in-situ* gel. The preformed gel formulations demonstrated shear thinning behaviour which is desirable for ease of handling and administration. The shear thinning behaviour is observed due to breaking of three-dimensional gel structures with increased rate of shear. With increase in poloxamer 188 concentration and increase in HPMC and chitosan concentration, there was increase in viscosity of formulation.

### Effect of dilution on gelling temperature 

After administration to nasal cavity, the gel may get diluted due to presence of nasal secretions. The gel should withstand the dilution effect and should retain its gelling temperature in the range of 30 to 35°C. The temperature less than 30°C could result in very viscous formulations difficult to handle and administer. The formulation with gelling temperature more than 35°C may result in improper gelling resulting in leakage or low retention. Therefore effect of dilution of gelling temperature of formulations was studied. With addition of phosphate buffer (pH 6.8) resulted in increase in gelation temperature. Formulation with poloxamer 188 (5% and 10%) and with lower concentration of mucoadhesive agent demonstrated gelation temperature in the favourable range (30-35°C). At 5% poloxamer188 concentration, chitosan (0.2% to 0.6%) was able to retain the gelling temperature in the range of 30-35°C with and without dilution.

### Effect on drug release 

Equation 4, 5 and 6 indicate effect of poloxamer188 concentration and mucoadhesive polymer concentration on drug release after 8h for *in-situ* gel formulations in presence of HPMC, HEC and chitosan respectively.The equations 4, 5 and 6 are given as following:

Y_2_= +42.457-9.2700*X_1_+1.790*X_3_+4.067*X_1_X_3_-6.388*X_1_^2^-4.486*X_3_^2^(4)

Y_2_= +45.202-7.99*X_1_+0.3175*X_3_+4.067*X_1_X_3_-6.388*X_1_^2^-4.486*X_3_^2^(5)

Y_2_= +43.59-7.835*X_1_-2.16000*X_3_+4.067*X_1_X_3_-6.388*X_1_^2^-4.486*X_3_^2^(6)

**Figure 8:**
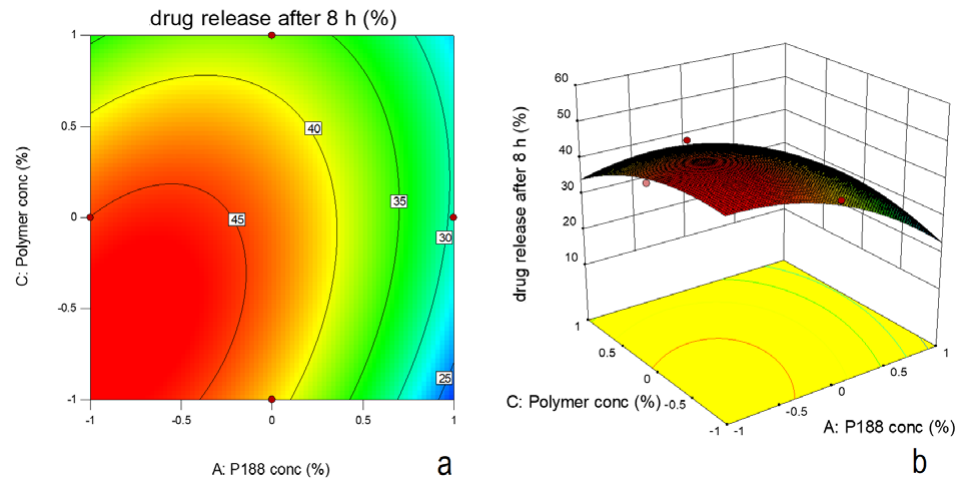
(a) Contour plot (b) Surface response plot depicting the effect of polymer concentration (chitosan) and poloxamer188 concentration on drug release.

**Figure 9:**
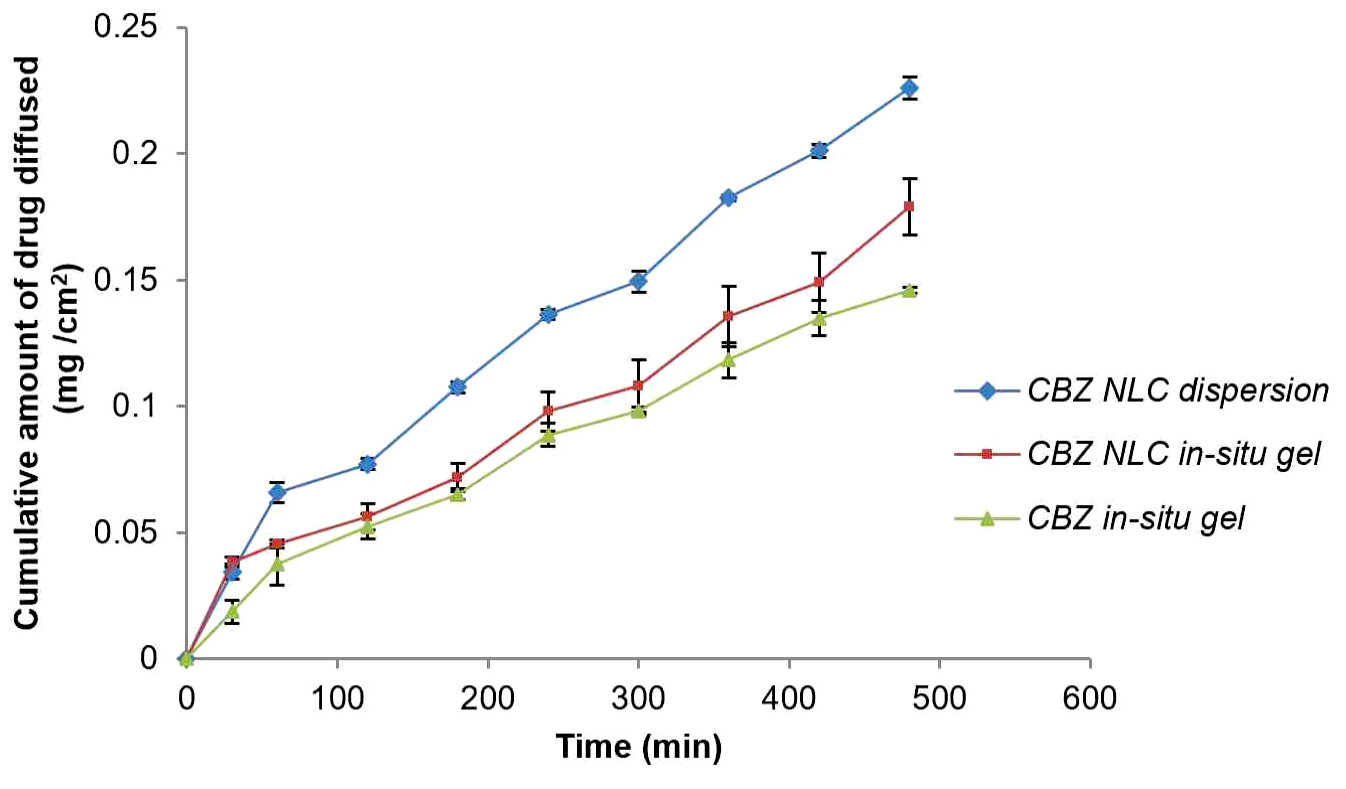
*In vitro* diffusion of nanostructured lipid carrier *in-situ* gel and plain *in-situ* gel.

Based on F-value (22.95) and p value (*P* ≤ 0.05), the model was observed as significant. Based on ANOVA data (Table 4), only poloxamer 188 concentration had significant effect on drug release and other two factors were insignificant. The interactive effect of X_1_ and X_3_ was also observed. The nonlinearity in the response was suggested by quadratic term (Figure 8a and b). Increase in poloxamer188 concentration in the formulation led to increase in viscosity of the formulation. Increased viscosity resulted in decrease in drug release from the formulation. Figure 9 indicates the comparison of diffusion profile of NLC *in-situ* gel with plain *in-situ* gel and NLC dispersion.

In order to study the drug release mechanisms from NLC based *in-situ* gel formulations, diffusion data was fitted to different dissolution kinetic models like zero order, first order, Higuchi matrix, Korsmeyer-Peppas, and Hixson Crowell (Table 5). Based on the values of R^2^ and the value of n of Korsmeyer-Peppas model, the drug release mechanism was confirmed as non-Fickian or anomalous.

**Table 5:** Release Kinetics of *in-situ* gelling formulations

**Code**	**Zero order**	**First order**	**Matrix**		**Korsmeyer-Peppas**		**Hixson Crowell**
	**R** ^ 2 ^	**R** ^ 2 ^	**R** ^ 2 ^	**K**	**R** ^ 2 ^	**n**	**R** ^ 2 ^
CBZ NLC dispersion	0.9814	0.9906	0.9700	18.89	0.9920	0.6834	0.9919
CBZ *in-situ* gel	0.9797	0.9920	0.9732	12.35	0.9936	0.6812	0.9892
CBZ NLC *in-situ* gel	0.9835	0.9861	0.9497	14.76	0.9540	0.6131	0.9875

Note. CBZ: Carbamazepine, NLC: Nanostructured lipid carriers, K is Higuchi constant and n value in Korsmeyer-Peppas indicates mechanism of drug release

### Mucoadhesive strength

**Figure 10:**
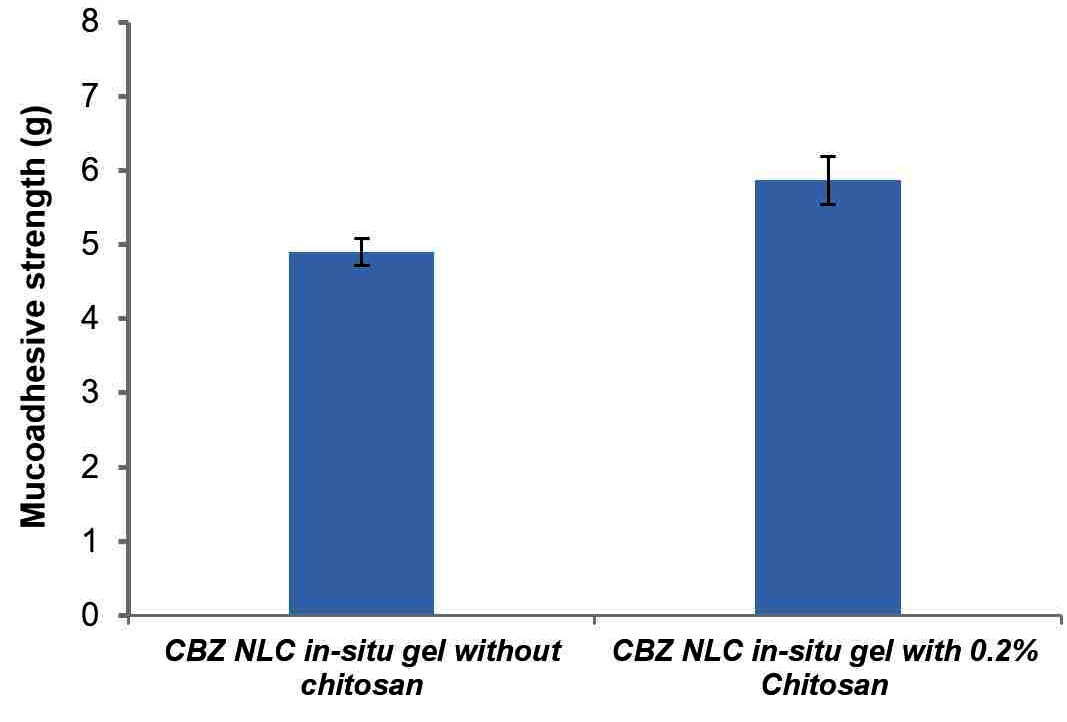
Mucoadhesive strength of carbamazepine nanostructured lipid carrier loaded *in-situ* gel.

Mucoadhesive strength determines the residence time of a formulation on mucosal surface; therefore it is an important parameter. The mucoadhesive strength of preformed gels (at 37°C) is represented in Figure 10. *In-situ* gelling formulation with chitosan represents higher mucoadhesion (5.8 g) on nasal mucosal surface than *in-situ* gel without chitosan. Mucoadhesion interaction is the result of polymer interaction to mucin via hydrogen and electrostatic interactions. Chitosan being a cationic polymer is reported to interact with negatively charged sialic acid moieties in the mucin resulting in higher mucoadhesion. Addition of NLC also contributed significantly to mucoadhesion by forming a closer contact of spherical nanoparticle on mucosal surface.

### Effect on ex-vivo diffusion through sheep nasal mucosa

**Figure 11:**
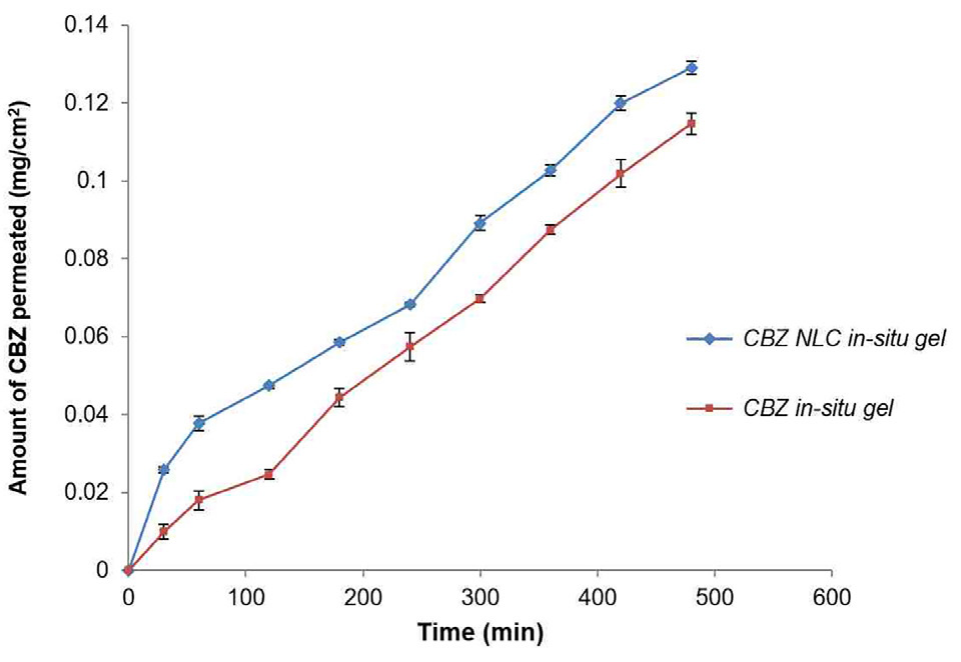
Ex- vivo diffusion of nanostructured lipid carrier based in-situ gel and plain *in-situ* gel through sheep nasal mucosa.

Figure 11 indicates nasal permeation profile of CBZ NLC loaded *in-situ* gel and CBZ plain gel without NLC. After 8 h diffusion study, the amount of CBZ permeated through nasal sheep mucosa from NLC based *in-situ* gel and plain *in-situ* gel formulation was 0.1291±0.001 mg/cm^2^ and 0.1146±0.002 mg/cm^2^ respectively.

The amount of CBZ permeated from CBZ NLC *in-situ* gel was significantly higher than plain CBZ *in-situ* gel as evident from the flux value, 0.0003±0.00001 mg. cm^-2^.h^-1^ and 0.0002±0.0001 mg.cm^-2^ h^-1^ respectively (Table 6). This is further observed from the higher permeability coefficient of NLC *in-situ* gel. The increase in CBZ permeation was 1.5 fold from NLC *in-situ* gel compared to plain *in-situ* gel. This could be due to close contact of NLC formulation with mucosal surface and higher mucoadhesion resulting in higher permeation rate.

**Table 6:** *Ex vivo* diffusion parameters

**Formulations**	**Flux (** ***Jss*** **± SD)** **(mg.cm**^-2^**.h**^-1^**)**	**Permeability coefficient** *** (*** ***Kp*** ***)*** **cm.h**^-1^	**Enhancement** **ratio (Er)**
CBZ NLC in-situ gel	0.0003±0.00001	0.0001	1.5
Plain CBZ in-situ gel	0.0002±0.0001	0.000061	

Note. Values are expressed as Mean ± SD (n=3), CBZ: Carbamazepine, NLC: Nanostructured lipid carriers Jss: Steady state Flux

### Anticonvulsant activity 

**Figure 12:**
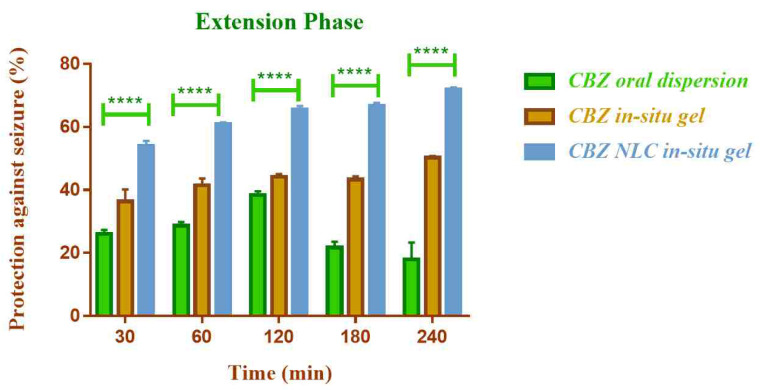
Comparison of *in vivo* anticonvulsant activity of formulations in extension phase (****indicates *P* ≤ 0.0001).

**Figure 13:**
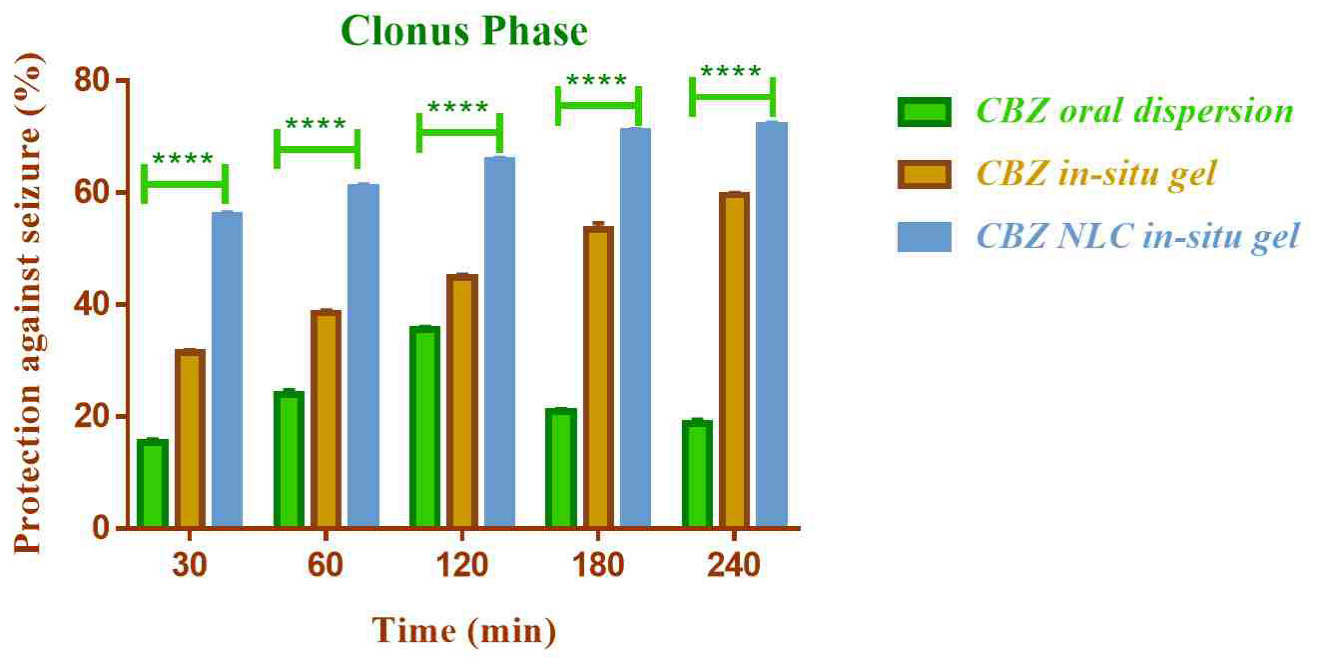
Comparison of *in vivo* anticonvulsant activity of formulations in clonus phase (****indicates *P* ≤ 0.0001).

The *in vivo* anticonvulsant activity of NLC loaded *in-situ* gelling formulation of CBZ was compared with CBZ *in-situ* gel (without NLC) by nasal route and oral CBZ dispersion by maximal electric seizure model in rats. The effect on the tonic and clonic phases of convulsion in rat was observed (Figure 12 and Figure 13).

The NLC based nasal *in-situ* gel demonstrated significantly higher anticonvulsant activity (*P* ≤0.0001) compared to plain nasal *in-situ* gel and oral dispersion. The percent protection against seizure with CBZ NLC *in-situ* gel formulation was 71.95% and for CBZ *in-situ* gel and CBZ oral dispersion was 50.26% and 13.22% respectively.

The lower activity of oral dispersion could be due to low solubility and lower bioavailability of CBZ by oral route. The efficacy was improved by nasal route and additionally the use of NLC enhanced the efficacy further. The higher anticonvulsant activity of NLC based formulation could be attributed to higher permeation of CBZ from NLC through nasal mucosa. The activity was found to be improved in both clonic and extension phase of seizure.

## Conclusion

In the present study, NLC based *in-situ* gelling intranasal formulation was developed for CBZ. The optimized formulation consisted of CBZ NLC incorporated into *in-situ* gel formulations prepared using poloxamer 407 and 188 along with chitosan as mucoadhesive polymer. The optimized formulation demonstrated excellent gelling ability, mucoadhesion, and gelling temperature in the range of 30-35°C and exhibited sustained drug release behaviour up to 8 hours. The drug diffusion through sheep nasal mucosa was higher from NLC based formulation. Improved *in vivo* anticonvulsant activity was demonstrated by MES model in rats compared to oral dispersion of CBZ and nasal CBZ *in-situ* gel without NLC. Conclusively, NLC based *in-situ* gelling intranasal formulation could be a potential approach for CBZ delivery with improved anticonvulsant activity.

## Ethical Issues

All the animal experiments conducted in the present study were approved by institutional animal ethical committee (Reg no: 198/PO/Re/S/2000/CPCSEA). The protocol approval number is DYPIPSR/IAEC/18-19/P-04. All the experiments were strictly adhering to guidelines of committee for the control and supervision of experiments on animals (CPCSEA), India.

## Conflict of Interest

Authors declare no conflict of interest.

## Acknowledgments

The authors are thankful to Gattefosse India Pvt Ltd for providing gift samples of Precirol ATO 5, Capryol 90 and GMS. Our sincere thanks to US Abitech Corp and BASF India for providing gift samples of Capmul MCM, Captex 500, Captex 200, Caprol PGE and Kolliphor P 407, Kolliphor P 188 respectively. Authors are also thankful to Amol organics Pvt Ltd, Vadodara, India for providing the sample of CBZ.
